# Indoleamine 2 3-dioxygenase knockout limits angiotensin II-induced aneurysm in low density lipoprotein receptor-deficient mice fed with high fat diet

**DOI:** 10.1371/journal.pone.0193737

**Published:** 2018-03-01

**Authors:** Sarvenaz Metghalchi, Marie Vandestienne, Yacine Haddad, Bruno Esposito, Julien Dairou, Alain Tedgui, Ziad Mallat, Stephane Potteaux, Soraya Taleb

**Affiliations:** 1 Institut National de la Santé et de la Recherche Médicale (Inserm), Paris Cardiovascular Research Center, and Université Paris-Descartes, Paris, France; 2 UMR 8601 CNRS, Laboratoire de Chimie et Biochimie Pharmacologiques et Toxicologiques, Université Paris Descartes-Sorbonne Paris Cité, Paris, France; 3 Division of Cardiovascular Medicine, University of Cambridge, Addenbrooke's Hospital, Cambridge, United Kingdom; Max Delbruck Centrum fur Molekulare Medizin Berlin Buch, GERMANY

## Abstract

**Aims:**

Abdominal aortic aneurysm (AAA) is an age-associated disease characterized by chronic inflammation, vascular cell apoptosis and metalloproteinase-mediated extracellular matrix degradation. Despite considerable progress in identifying targets involved in these processes, therapeutic approaches aiming to reduce aneurysm growth and rupture are still scarce.

Indoleamine 2–3 dioxygenase 1 (IDO) is the first and rate-limiting enzyme involved in the conversion of tryptophan (Trp) into kynurenine (Kyn) pathway. In this study, we investigated the role of IDO in two different models of AAA in mice.

**Methods and results:**

Mice with deficiencies in both low density receptor-deficient (*Ldlr*^*-/-*^) and IDO (*Ldlr*^*-/-*^*Ido1*^*-/-*^) were generated by cross-breeding *Ido1*^*-/-*^ mice with *Ldlr*^*-/-*^mice. To induce aneurysm, these mice were infused with angiotensin II (Ang II) (1000 ng/min/kg) and fed with high fat diet (HFD) during 28 days. AAAs were present in almost all *Ldlr*^*-/-*^ infused with AngII, but only in 50% of *Ldlr*^*-/-*^*Ido1*^*-/-*^ mice. Immunohistochemistry at an early time point (day 7) revealed no changes in macrophage and T lymphocyte infiltration within the vessel wall, but showed reduced apoptosis, as assessed by TUNEL assay, and increased α-actin staining within the media of *Ldlr*^*-/-*^*Ido1*^*-/-*^ mice, suggesting enhanced survival of vascular smooth muscle cells (VSMCs) in the absence of IDO. In another model of elastase-induced AAA in C57Bl/6 mice, IDO deficiency had no effect on aneurysm formation.

**Conclusion:**

Our study showed that the knockout of IDO prevented VSMC apoptosis in AngII -treated *Ldlr*^*-/-*^ mice fed with HFD, suggesting a detrimental role of IDO in AAA formation and thus would be an important target for the treatment of aneurysm.

## Introduction

Abdominal aortic aneurysm (AAA) is an age-associated disease with increasing incidence and considerable consequences on morbidity and mortality [1]. Mechanistically, AAA is characterized by vessel dilatation due to medial degeneration with elastin degradation, collagen remodeling, vascular smooth muscle cell (VSMC) apoptosis and chronic aortic wall inflammation [2]. Matrix remodeling is conditioned by the imbalance between metalloproteinases (MMP) and Tissue Inhibitor of Metalloproteinases (TIMP) [3]. However, until now, open surgical repair appears as the only accessible approach, as no therapeutic targets have been yet identified to prevent aneurysm growth or rupture.

Indoleamine 2 3- dioxygenase 1 (IDO) is an enzyme that catalyzes the degradation of the essential amino acid L-tryptophan (Trp) to N-formylkynurenine leading to the generation of several active metabolites constituting the kynurenine (Kyn) pathway [4]. Recently, experimental studies showed that IDO was involved in the pathogenesis of atherosclerosis [5, 6], and its plasma activity was associated with worse cardiovascular outcome in coronary patients [6–9]. Angiotensin (Ang) II, widely used to induce dissecting aortic aneurysm [10], was previously shown to increase IDO activity through the production of mitochondrial reactive oxygen species [11]. Moreover, a recent report indicates that IDO deficiency in AngII-infused apolipoprotein e-deficient (*Apoe*^*-/-*^) mice protects against AAA[12]. The authors attributed the pro-aneurysmal effect of IDO to one of the Kyn-derived metabolites, 3-hydroxyanthranilic acid (3-HAA), which induced aneurysm by increasing MMP-2 expression in VSMC[12].

High fat diet (HFD) containing cholesterol has been shown to accelerate AngII-induced aneurysm formation in mice [13, 14], which mimics some aspects of aneurysmal human disease [15]. In this study, we investigate the role of IDO in two murine models of AAA using low density receptor-deficient (*Ldlr*^*-/-*^) mice fed a HFD during 28 days, either after infusion of AngII (dissecting AAA) or after topical peri-aortic elastase (non-dissecting AAA)

## Materials and methods

### Mice and aneurysm models

Experiments were conducted according to the guidelines formulated by the European Community for experimental animal use (Directive 2010/63/EU) and were approved by the Ethics Committee of INSERM and the French Ministry of Agriculture (agreement A75-15-32). All experiments were conducted on male mice between 8 and 12 weeks of age. *C57Bl/6 Ido1*^*-/-*^ and *Ldlr*^*-/-*^ mice were from the Jackson Laboratory. *Ldlr*^*-/-*^*Ido1*^*-/-*^ mice were obtained by crossing *Ido1*^*-/-*^ and *Ldlr*^*-/-*^ mice.

AAA was induced in hypercholesterolemic mice as previously described in[10]. AAA was induced in *Ldlr*^*-/-*^ and *Ldlr*^*-/-*^ *Ido1*^*-/-*^ mice by subcutaneous infusion of AngII (Sigma-Aldrich) at 1μg/kg/min and high fat diet (HFD) containing 15% fat, 1.25% cholesterol, and 0% cholate for 7 days or 28 days. Angiotensin II (Ang II) was purchased from Sigma-Aldrich (St. Louis, MO, USA), and ALZET osmotic pumps (model 2004) were from Charles River Laboratories. A 5-point grading system was used to classify aneurysms based on Daugherty et al classification [16]. Type 0: Normal aorta. Type I, dilated lumen in the supra-renal region of the aorta with no thrombus. Type II, remodeled tissue in the supra-renal region that frequently contains thrombus. Type III, a pronounced bulbous form of Type II that contains thrombus. Type IV, a form in which there are multiple aneurysms containing thrombus, some overlapping, in the suprarenal area of the aorta or rupture.

The second model of AAA was performed as previously reported[17]. Briefly, normocholesterolemic *C57Bl/6J* and *C57Bl/6 Ido1*^*-/-*^ were anesthetized with 2% Isoflurane, placed on a heating pad and received 0.05 mg/kg subcutaneous (s.c.) buprenorphine. After median laparotomy, the abdominal aorta, from the left renal vein to the iliac bifurcation, was exposed and bathed in 10 μL of filtered porcine pancreatic elastase (E1250; Sigma-Aldrich) during 5 min. The aorta was flushed three times with 0.9% NaCl to stop reaction.

### Characterization of aneurysmal lesions

The suprarenal region of the abdominal aorta containing AAAs was serially cross-sectioned (8 μm sections). Paraffin embedded aortas were used for different stainings. Elastin staining was visualized using Orcein, the mean number of elastin layers was quantified for each mice. The extent of vascular smooth muscle cells (VSMC) was evaluated using anti-αSMC (Sigma-Aldrich) within media. T lymphocytes were detected using anti-CD3 antibody (Dako), macrophages using anti-CD68 antibody (AbD serotec). IHC chromogen substrate AEC (Thermo scientific) was used for revelation. The extent of apoptosis cells was evaluated by Terminal dUTP nick end-labelling (TUNEL) staining, using In Situ Cell Death Detection Kit (Sigma-Aldrich). We performed morphometric studies using Histolab software (Microvisions).

### Flow cytometry

Flow cytometry staining was performed at day 7 after AngII infusion. Monocytes were identified as CD11b+CD115+, classical monocytes CD11b+CD115+GR-1high and nonclassical monocytes CD11b+CD115+GR-1low. Stainings included the following antibodies: V-450 or FITC-conjugated anti-CD11b (M1/70, BD Biosciences) PE-conjugated anti-CD115 (AFS 98, eBioscience) Anti-Gr1 (Ly6C and G)-PERCP-Cy5 (RB6-8C5, BD Biosciences), anti-CD4- V-450 (RM4-5, eBioscience) anti-CD3ε- APC (145-2C11, eBioscience), anti-CD8a-AF-700 (53–6.7, BD Biosciences) anti-MHCII- FITC (M5/114.15.2, eBioscience) anti-CD19-AF-700 (6D5, BD Biosciences) and APC-conjugated CD25 (PC61.5). Intracellular staining of forkhead box P3 (PE or PECy 7-conjugated FOXP3) (eBiosciences) was performed. For intracellular cytokine staining, splenocytes were stimulated *in vitro* for 4 h using leukocyte activation cocktail (BD) and with lipopolysaccharide (LPS) at 1 ug/ml for IL-10 staining. Briefly, cells were stained for surface markers followed by fixation and permeabilization using a kit (eBiosciences) for intracellular staining. Then, cells were stained with Brilliant Violet 421-conjugated Ifn-γ (clone XMG1.2), PE-conjugated Il-17 (clone TC11-18H10.1) (BioLegend) and APC-conjugated Il10 (clone JES5-16E3) (eBiosciences).

Forward scatter (FSC) and side scatter (SSC) were used to gate live cell excluding red blood cells, debris, and cell aggregates in total blood cells and splenocytes. Cells were acquired using a BD LSRII Fortessa flow cytometer (BD Biosciences) and analyzed with FlowJo (Tree Star, Inc.).

### HPLC measurements

100 μL of the sample were deproteinized by 50 μl of 15% perchloric acid. The proteins were pelleted by centrifugation at 13,000 rpm for 30 minutes. Separation of Kyn and Trp was done by reversed-phase liquid chromatography using a 20mM NaH2PO4 buffer (not pH adjusted) with 5.0% acetonitrile. The mobile phase was delivered by an HPLC pump (Shimadzu, Japan) through a Supelcosil C18 column (250 mm x 4.6 mm, 5 μm, Supelco, USA) at a rate of 1 ml/min. Following separation, the analysate was first passed through a guard cell with an oxidizing potential of 50 mV. Samples were then quantified by sequential oxidation and reduction in a high-sensitivity analytical cell (ESA 5010; ESA Inc, USA) controlled by a potentiostat (Coulochem III; ESA Inc, USA) with an applied potential of 600 mV and 700 mV for detection of Kyn and Trp. The signals from the detector were transferred to a computer for analysis (Labsolution, Shimadzu). The retention time of Kyn was approximately 12 min and approximately 21 min for Trp.

10–20 mg of aorte samples were lyzed and deproteinized by 150 μl of 7.5% perchloric acid. Lysis was complete by the sonication and the proteins were pelleted by centrifugation at 13,000 rpm for 30 minutes. Separation of Kyn and Trp was done by reversed-phase liquid chromatography using a 20mM NaH2PO4 buffer (not pH adjusted) with 5.0% acetonitrile. The mobile phase was delivered by an HPLC pump (Shimadzu, Japan) through a Supelcosil C18 column (250 mm x 4.6 mm, 5 μm, Supelco, USA) at a rate of 1 ml/min. Following separation, the analysate was first passed through a guard cell with an oxidizing potential of 50 mV. Samples were then quantified by sequential oxidation and reduction in a high-sensitivity analytical cell (ESA 5010; ESA Inc, USA) controlled by a potentiostat (Coulochem III; ESA Inc, USA) with an applied potential of 600 mV and 700 mV for detection of Kyn and Trp. The signals from the detector were transferred to a computer for analysis (Labsolution, Shimadzu). The retention time of Kyn was approximately 13 min and approximately 17.5 min for Trp. The sensitivity of the system was verified by analysis of standard mixtures of Kyn, with concentrations from 50 to 1000nM, and Trp, with concentrations from 0.5 to 50 μM, resulting in a linear standard plot.

### Quantitative real time polymerase chain reaction

The abdominal aorta was cleared of all the surrounding tissues. Tissue samples were pulverised using IKA™ T 25 digital homogenizer (Thermo Fisher Scientific). Total RNA was extracted from tissue samples using Trizol reagent (Invitrogen). Isolated RNA was reverse-transcribed using Quantitect Reverse Transcription kit (Qiagen). Real-time PCR was performed on cDNA product using Takyon qPCR mix on an Step-One Plus (Applied Biosystems) in duplicates. GAPDH was used to normalize gene expression. The following primer sequences were used:

Gapdh (F: 5′-CGT CCC GTA GAC AAA ATG GTG AA-3′; R: 5′-GCC GTG AGT GGA GTC ATA CTG GAA CA-3′);

TIMP-1 (F: 5′-CCC CCT TTG CAT CTC TGG CAT CT-3′; R: 5′-GCG GTT CTG GGA CTT GTG GGC ATA-3′);

TIMP-2 (F: 5′-GGC-CCC-CTC-TTC-AGG-AGT-3′; R: 5′-TCC-CAG-GGC-ACA-ATG-AAG-T-3′);

TIMP-3 (F: 5′-GGC-CTC-AAT-TAC-CGC-TAC-CAC-3′; R: 5′-GGC-GTT-GCT-GAT-GCT-TTC-GT-3′);

MMP-2 (F: 5′-CCG AGA CCG CTA TGT CCA CTG T-3′; R: 5′-CCG GTC ATC ATC GTA GTT GGT TGT-3′);

MMP-9 (F: 5′-CCG TCA TTC GCG TGG ATA AGG AGT-3′; R: 5′-GTA GCC CAC GTC CAC CTG GTT-3′);

MMP-12 (F: 5′-CCC CCA TCC TTG ACA AAA CCT-3′; R: 5′-TGG CGA AGT GGG TCA AAGA-3′). Relative expression was calculated using the 2-delta-delta CT method.

### *Ex vivo* reflectance epifluorescence imaging

Mice were anaesthetized with isoflurane and received intravenously 150 μL of a fluorescent imaging probe MMPsense 680 (NEV 10126, PerkinElmer) 24 h before sacrifice. This agent is optically silent in its unactivated state and becomes highly fluorescent following activation by MMPs including MMP-2, -3, -9, and -13. Images were acquired using a fluorescence molecular imaging system (FMT 2500TM, VisEn Medical).

### Statistics

Values are expressed as means ± s.e.m. Differences between values were examined using Mann Whitney test and χ^2^ test for a trend. Values were considered significant at *P*≤0.05.

## Results and discussion

### IDO deficiency protects against AngII-induced AAA in *Ldlr*^*-/-*^ mice fed a HFD

IDO is involved in the conversion of Trp to Kyn. We then examined the effects of HFD and AngII on plasma Trp and Kyn in *Ldlr*^*-/-*^ mice. As shown in **S1 Fig**, after 28 days of HFD alone, no significant increase of Kyn was observed in the plasma of *Ldlr*^*-/-*^ mice (P = 0.14). However, in agreement with a previous study [11], AngII infusion (1000 ng/min/kg) significantly increased plasma Kyn without Trp changes in *Ldlr*^*-/-*^ mice after 1 week of treatment (P = 0.02), suggesting that AngII stimulated IDO activity.

To study the role of IDO in aneurysm, we generated double knockout *Ldlr*^*-/-*^*Ido1*^*-/-*^ mice, and put *Ldlr*^*-/-*^*Ido1*^*+/+*^ and *Ldlr*^*-/-*^*Ido1*^*-/-*^ mice on HFD with Ang II infusion (1000 ng/min/kg) during 28 days.

**Fig 1:**
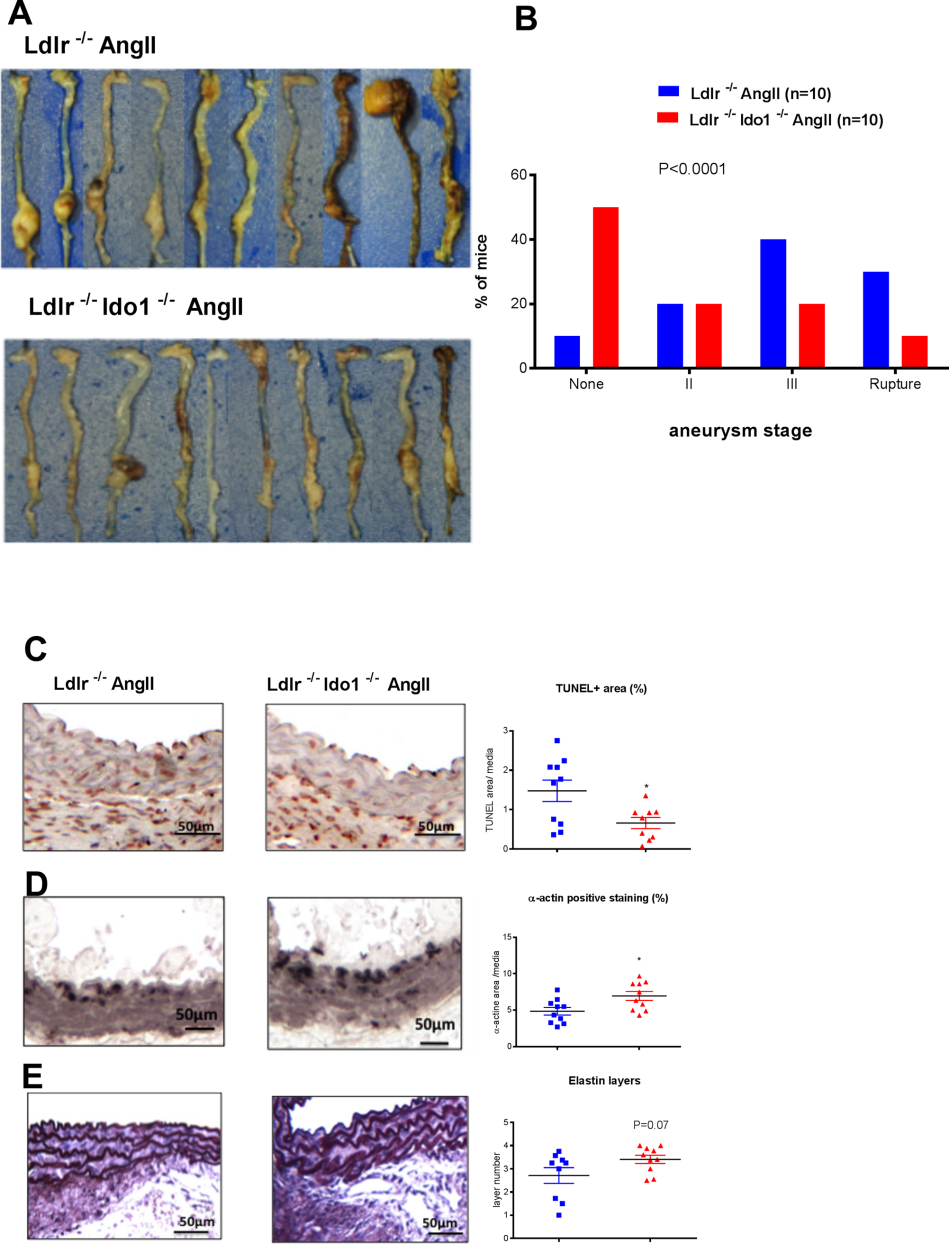
IDO deficiency protects against AAA in AngII-induced aneurysm in *Ldlr*^*-/-*^ mice fed a high fat diet. **A-B** Images and classification of aortic aneurysm in *Ldlr*^*-/-*^ (n = 10) and *Ldlr*^*-/-*^*Ido1*^*-/-*^ (n = 10) mice infused with AngII (1000ng/min/kg) and fed a high fat diet (HFD) during 28 days. The results were confirmed in two independent experiments (n = 10 per group). **C-E** Representative images and quantifications of apoptotic area assessed by TUNEL assay (**C**), α-actin staining (**D**) and elastin layers visualized by Orcein coloration (**E**) in *Ldlr*^*-/-*^ (n = 10) and *Ldlr*^*-/-*^*Ido1*^*-/-*^ (n = 10) mice infused with AngII (1000ng/min/kg) and fed HFD for 28 days. Mean values ± SEM are shown. *P≤0.05.

IDO was previously shown to contribute to vessel relaxation in lipopolysaccharide-induced endotoxin shock[18]. We thus determined systolic blood pressure levels in *Ldlr*^*-/-*^*Ido1*^*+/+*^ and *Ldlr*^*-/-*^*Ido1*^*-/-*^ mice subjected to AngII and HFD at day 0, 5, and 14. As expected, Ang II significantly increased systolic blood pressure in *Ldlr*^*-/-*^*Ido1*^*+/+*^ mice, but the increase was similar in *Ldlr*^*-/-*^*Ido1*^*-/-*^ mice (**S2 Fig**), indicating that IDO was not involved in the regulation of systolic blood pressure in our model. As depicted in **Fig 1A and 1B,** the absence of IDO protected against AngII-induced aneurysm formation and severity (P<0.0001). This protective effect was associated with significantly reduced TUNEL positive area (p = 0.05) (**Fig 1C)** and increased α-actin staining (p = 0.03) (**Fig 1D**), as well as a trend towards more elastic layers within the media (p = 0.07) (**Fig 1E**).

To investigate the mechanisms involved in AAA formation, we implanted *Ldlr*^*-/-*^*Ido1*^*+/+*^ and *Ldlr*^*-/-*^*Ido1*^*-/-*^ mice with pumps infusing either PBS or Ang II, and then put the mice on HFD for a short duration (7 days). Inflammation was previously shown to be involved in aneurysm pathogenesis [19]. We thus examined whether changes in inflammatory responses may account for our findings.

As shown in **S3 Fig**, we found no significant differences in circulating neutrophils, classical and non-classical monocytes, CD4^+^ and CD8^+^ T cells, and CD19^+^ B cells in PBS-infused *Ldlr*^*-/-*^*Ido1*^*+/+*^ compared to *Ldlr*^*-/-*^*Ido1*^*-/-*^ mice fed with HFD. Production of spleen IFN-γ and IL-10 was unchanged, but IL-17 production was increased in PBS-infused *Ldlr*^*-/-*^*Ido1*^*-/-*^ compared to *Ldlr*^*-/-*^*Ido1*^*+/+*^ mice (**S3 Fig**).

**Fig 2:**
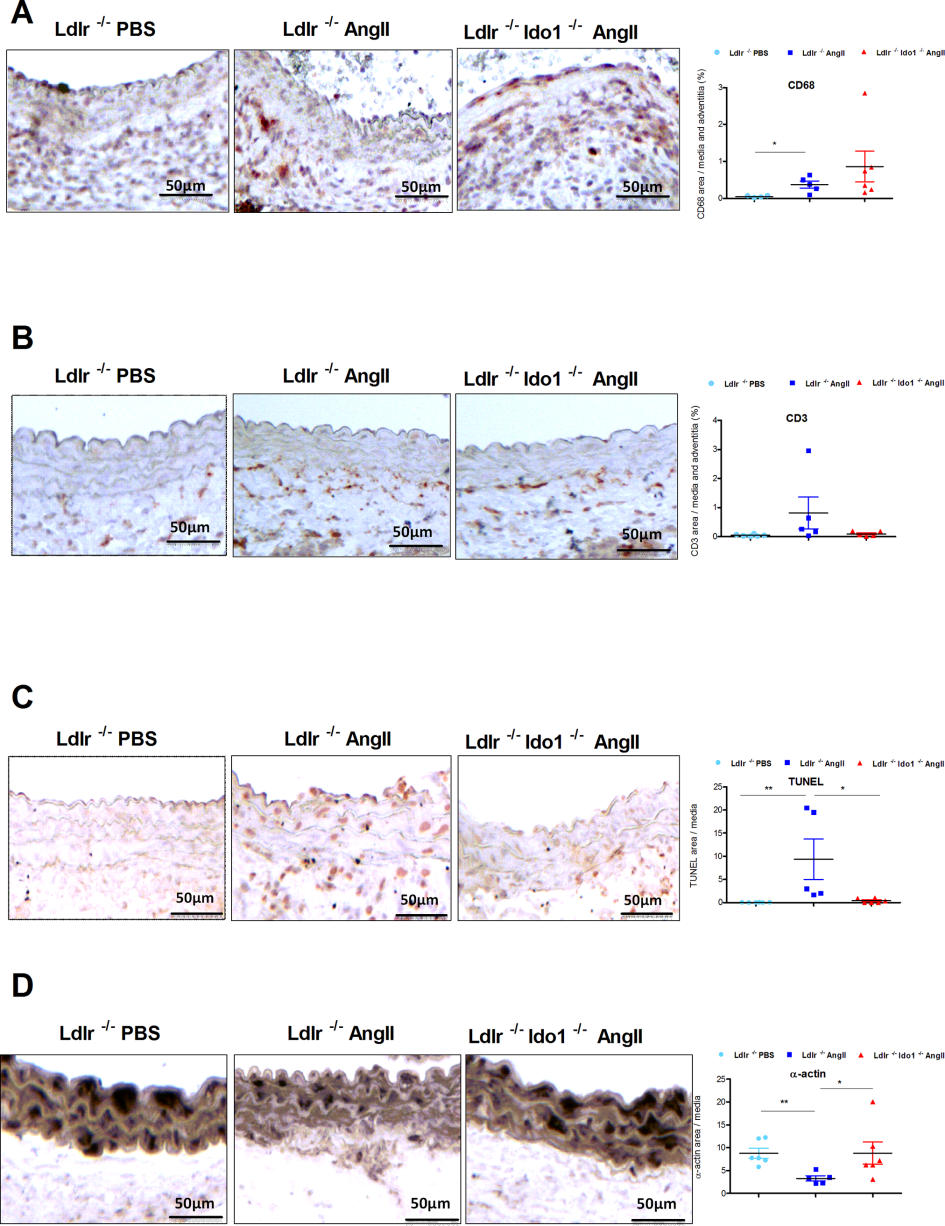
IDO deficiency prevents AngII-induced vascular smooth muscle cell apoptosis. **A-D** Representative images and quantifications of macrophages (CD68 staining) (**A**) T lymphocytes (CD3 staining) (**B**), apoptotic area as assessed by TUNEL assay (**C**) and α-actin (**D**) in *Ldlr*^*-/-*^ infused either PBS (n = 5), AngII (n = 5) and *Ldlr*^*-/-*^*Ido1*^*-/-*^ mice infused with AngII (n = 5) for 7 days. Mean values ± SEM are shown. *P≤0.05, **P<0.001.

Of note, in AngII-infused *Ldlr*^*-/-*^ group one mouse died before day 7 because of aneurysm rupture. No significant differences were observed in circulating neutrophils, classical and non-classical monocytes, CD4^+^, CD8^+^ T cells, CD8^+^, and CD19^+^ B cells in Ang II-infused *Ldlr*^*-/-*^*Ido1*^*+/+*^ compared to *Ldlr*^*-/-*^*Ido1*^*-/-*^ mice fed with HFD (**S4 Fig**). A significant decrease in protective T regulatory cells [20] was observed in the spleens of AngII-infused *Ldlr*^*-/-*^*Ido1*^*-/-*^ compared to *Ldlr*^*-/-*^*Ido1*^*+/+*^ mice (P = 0.002), despite a protection against aneurysm in absence of IDO. However, no significant differences were detected in IL-17, IL-10 and interferon (IFN)- γ production by splenic lymphocytes of the 2 groups of mice (**S4 Fig**). Aneurysm formation was associated with an accumulation of macrophages and T lymphocytes within the adventitia and the media, but no significant differences were observed between AngII-infused *Ldlr*^*-/-*^*Ido1*^*+/+*^ and *Ldlr*^*-/-*^*Ido1*^*-/-*^ mice (**Fig 2A and 2B**). A recent study reported that IDO promoted aneurysm formation through 3HAA production, which activated MMP2 expression[12]. Therefore, we investigated MMP expression and activity in our model. As shown in **S5 Fig**, we found no differences in mRNA expression of MMP-2, -9 and -12, and no differences in TIMP-1, -2, and -3 in the abdominal aorta isolated from AngII-infused *Ldlr*^*-/-*^*Ido1*^*+/+*^ compared to *Ldlr*^*-/-*^*Ido1*^*-/-*^ mice. Moreover, MMP protease activity in the aortic wall, quantified using *ex vivo* reflectance epifluorescence imaging, was similar in AngII-infused *Ldlr*^*-/-*^*Ido1*^*+/+*^ and *Ldlr*^*-/-*^*Ido1*^*-/-*^ mice, indicating that IDO is unlikely to be involved in MMP expression or activity in this model (**S5 Fig**). Interestingly however, AngII-induced TUNEL positivity within the media of AngII-infused *Ldlr*^*-/-*^ mice was prevented in the absence of IDO (P = 0.04) (**Fig 2C**), suggesting a protection of VSMC against apoptosis. In agreement with this observation, increased α-actin staining was observed within the media of AngII-infused *Ldlr*^*-/-*^*Ido1*^*-/-*^ compared to *Ldlr*^*-/-*^ mice (P = 0.03) (**Fig 2D**). In agreement with this result, IDO activity, through the generation of 3-hydroxykynurenine, has been shown to promote endothelial cell apoptosis in vitro [11].

### IDO deficiency does not alter aneurysm formation after elastase application

**Fig 3:**
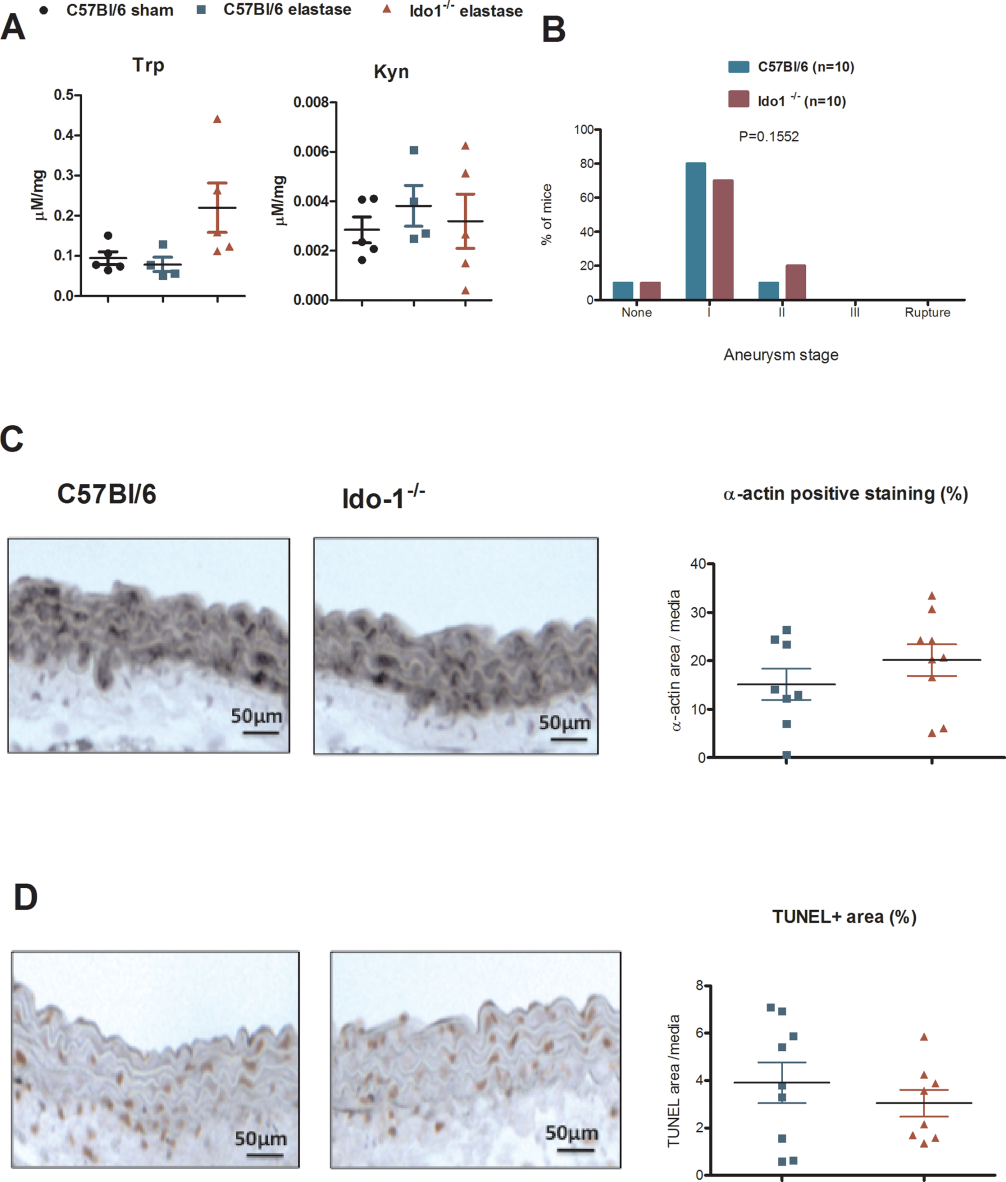
IDO deficiency has no effects in elastase-induced aneurysm model. **A** Trp and Kyn levels in aorta of C57Bl/6 mice sham group or C57Bl/6 or Ido-1^-/-^ mice treated with elastase (n = 4/per group) at day 3. **B-D** classification of aortic aneurysm in C57Bl/6 (n = 10) and Ido1^-/-^ mice (n = 10) treated with elastase **(B)**, α-actin staining (**C**) and TUNEL area (**D**) at day 3 after elastase treatment in C57Bl/6 (n = 10) and Ido1^-/-^ mice (n = 10).

We also tested the impact of IDO in a non-dissecting model of AAA induced by local elastase application. The latter directly degrades elastin, leading to VSMC apoptosis and inflammation within the media [21]. This model induces aortic dilatation without the need for hypercholesterolemia. Elastase-induced AAA formation did not induce Kyn production in aorta and was not affected by IDO deficiency nor were VSMC apoptosis (TUNEL-positive area), α-actin staining (**Fig 3**), and infiltration of T lymphocytes and macrophages (data not shown).

Taken together, our results show that IDO deletion protects against AAA formation in HFD-fed *Ldlr*^*-/-*^ mice by preventing AngII-induced VSMC apoptosis, but had no effect in AngII-independent elastase-induced AAA formation.

## Supporting information

S1 Fig AngII increases kynurenine in plasma of Ldlr^-/-^ mice. **A-B** plasma tryptophan (Trp) and Kynurenine (Kyn) in *Ldlr*^*-/-*^ mice at baseline (n = 10), after 4 weeks of HFD (n = 10), 1 week after AngII infusion (n = 10) and *Ldlr*^*-/-*^*Ido1*^*-/*-^ mice 1 week after AngII infusion (n = 5).
(PDF)

S2 Fig IDO deficiency has no effects on systolic blood pressure. Systolic blood pressure in *Ldlr*^*-/-*^ (n = 10) and *Ldlr*^*-/-*^*Ido1*^*-/-*^ (n = 10) mice fed with HFD at baseline, 5 days and 14 days after AngII infusion.
(PDF)

S3 Fig IDO deficiency effects in Ldlr^-/-^ infused with PBS and fed with HFD. **A** quantitative analysis of flow cytometry staining of blood neutrophils (CD11b+GR-1+), monocytes (CD11b+CD115+) and subsets, classical (GR1 high) and non classical (GR1 low) monocytes (**B**), B lymphocytes (CD19+) (**C**), T lymphocytes (CD4+, CD8+) (**D**) and T regulatory cells (CD25+Foxp3+) gated on CD4+ cells (**E**) in *Ldlr*^*-/-*^ and *Ldlr*^*-/-*^*Ido1*^*-/-*^ mice (n = 4/group) infused with PBS and fed with HFD during 7 days. **F** quantitative analysis of flow cytometry-based intracellular staining of interleukin (IL)-17, IL-10 and interferon (IFN)- γ gated on splenocytes in the 2 groups of mice.
(PDF)

S4 Fig IDO deficiency effects in Ldlr^-/-^ infused with AngII and fed with HFD. **A** quantitative analysis of flow cytometry staining of blood monocytes (CD11b+CD115+) and subsets, classical (GR1 high) and non classical (GR1 low) monocytes, neutrophils (CD11b+GR-1+), T lymphocytes (CD4+, CD8+) and B lymphocytes (CD19+) cells in *Ldlr*^*-/-*^ mice infused with either PBS (n = 5) or Ang II (n = 5) and *Ldlr*^*-/-*^*Ido1*^*-/-*^ mice infused with AngII (n = 5) and fed with HFD during 7 days. **B** representative pictures and quantifications of T regulatory cells (CD25+Foxp3+) gated on CD4+ cells. **C** quantitative analysis of flow cytometry-based intracellular staining of interleukin (IL)-17, IL-10 and interferon (IFN)-γ gated on splenocytes in the 3 groups of mice.
(PDF)

S5 Fig IDO deletion has no effects on MMP expression and activity. **A** MMP 2, 9 and 12 and TIMP1, 2, and 3 mRNA in aorta of *Ldlr*^*-/-*^ mice infused with either PBS (n = 5) or AngII (n = 5) and *Ldlr*^*-/-*^ *Ido-1*^*-/-*^ (n = 5) mice infused with Ang II and fed with HFD during 7 days. **B** Quantification of matrix metalloproteinase (MMP)-sense 680 activity in the abdominal and thoracic aorta, measured by ex vivo reflectance epifluorescence imaging in AngII-infused Ldlr^-/-^ and Ldlr^-/-^ Ido1^-/-^ mice (n = 5/group) fed with HFD during 7days.
(PDF)
