# Supplementary figures and images for: Indoleamine 2 3-dioxygenase knockout limits angiotensin II-induced aneurysm in low density lipoprotein receptor-deficient mice fed with high fat diet
Source: PLoS One. 2018 Mar 1;13(3):e0193737. doi: 10.1371/journal.pone.0193737 (PMC5833272; doi:10.1371/journal.pone.0193737)

**A****Plasma Trp**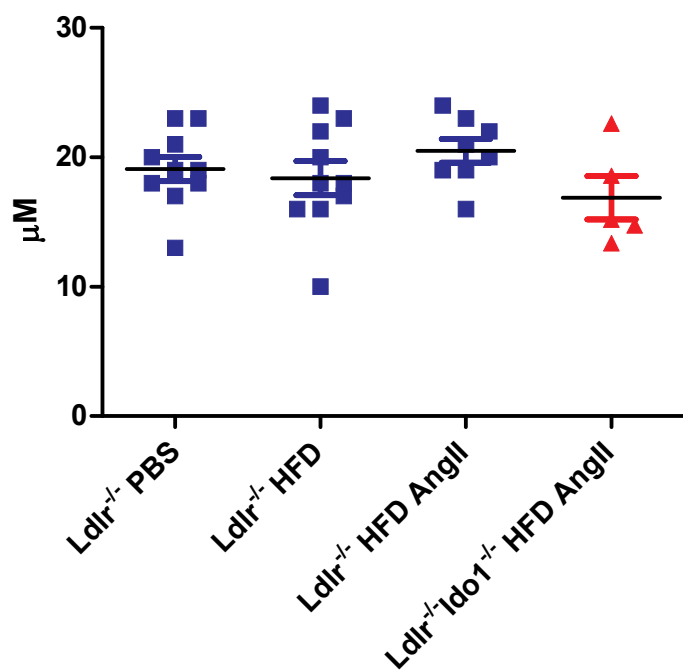**B****Plasma Kyn**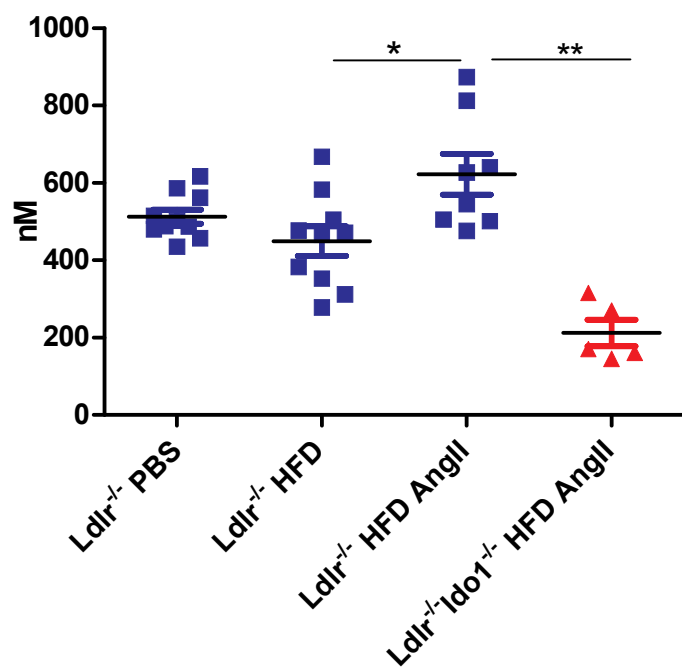**S1Fig.**

Supplement: S1 Fig — A-B plasma tryptophan (Trp) and Kynurenine (Kyn) in Ldlr-/- mice at baseline (n = 10), after 4 weeks of HFD (n = 10), 1 week after AngII infusion (n = 10) and Ldlr-/-Ido1-/- mice 1 week after AngII infusion (n = 5). (PDF) [file pone.0193737.s001.pdf]

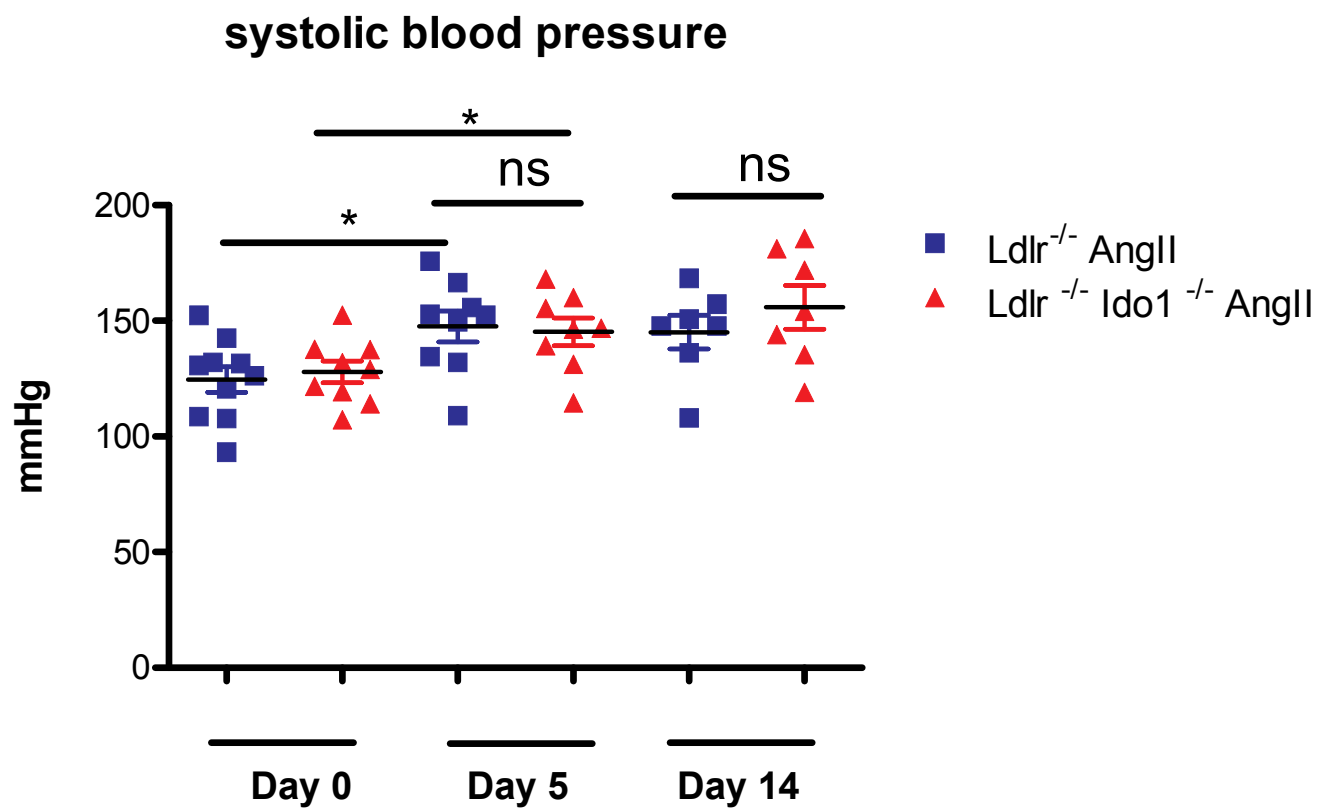

**S2Fig.**

Supplement: S2 Fig — Systolic blood pressure in Ldlr-/- (n = 10) and Ldlr-/-Ido1-/- (n = 10) mice fed with HFD at baseline, 5 days and 14 days after AngII infusion. (PDF) [file pone.0193737.s002.pdf]

**A**

●  $Ldlr^{-/-}$  PBS      ▲  $Ldlr^{-/-}$   $Ido1^{-/-}$  PBS

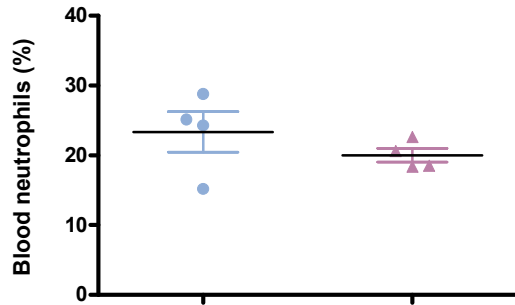**B**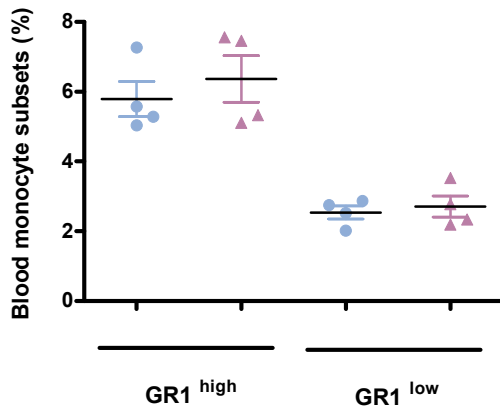**C**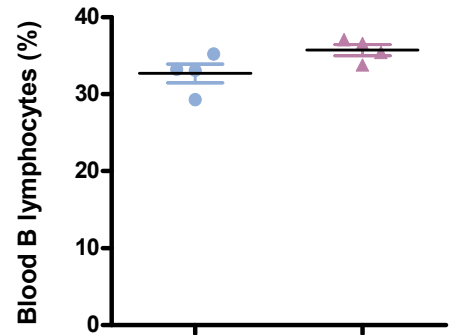**D**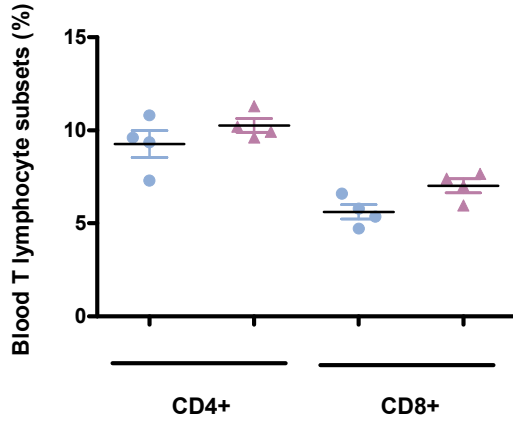**E**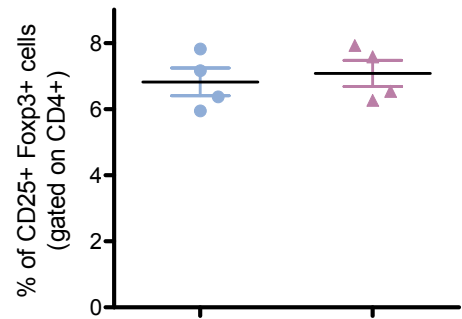**F**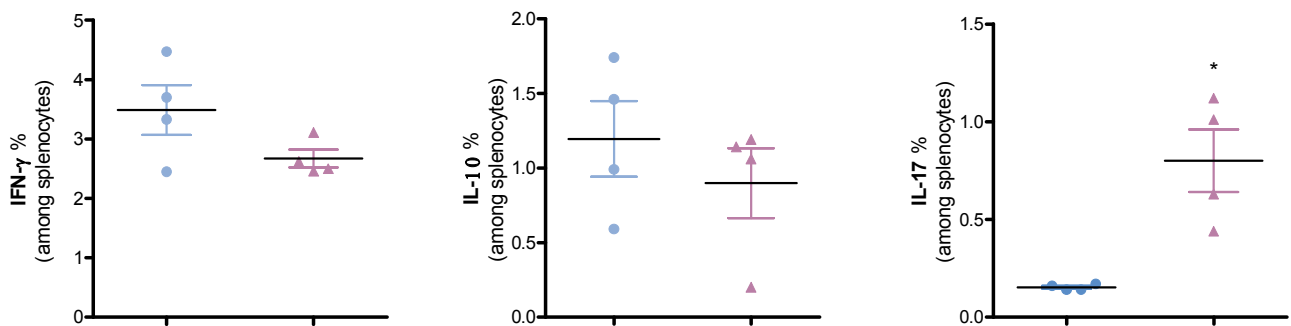

Supplement: S3 Fig — A quantitative analysis of flow cytometry staining of blood neutrophils (CD11b+GR-1+), monocytes (CD11b+CD115+) and subsets, classical (GR1 high) and non classical (GR1 low) monocytes (B), B lymphocytes (CD19+) (C), T lymphocytes (CD4+, CD8+) (D) and T regulatory cells (CD25+Foxp3+) gated on CD4+ cells (E) in Ldlr-/- and Ldlr-/-Ido1-/- mice (n = 4/group) infused with PBS and fed with HFD during 7 days. F quantitative analysis of flow cytometry-based intracellular staining of interleukin (IL)-17, IL-10 and interferon (IFN)- γ gated on splenocytes in the 2 groups of mice. (PDF) [file pone.0193737.s003.pdf]

**A**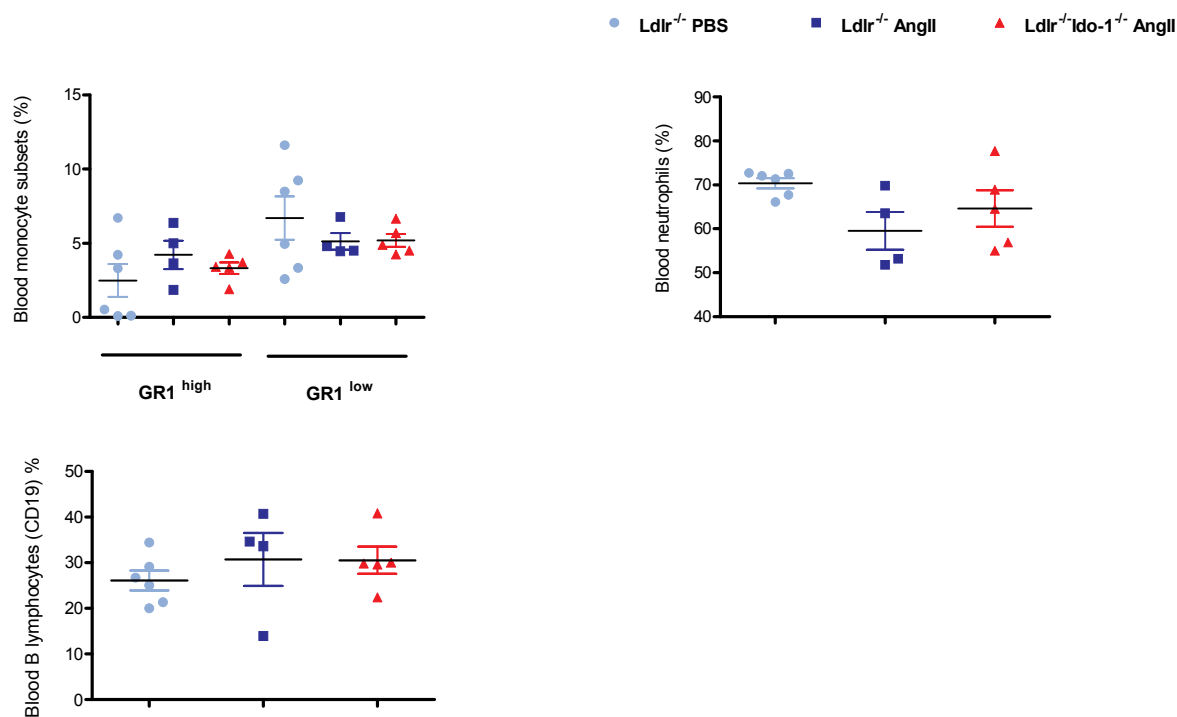**B** $Ldlr^{-/-}$  AngII $Ldlr^{-/-}Ido1^{-/-}$  AngII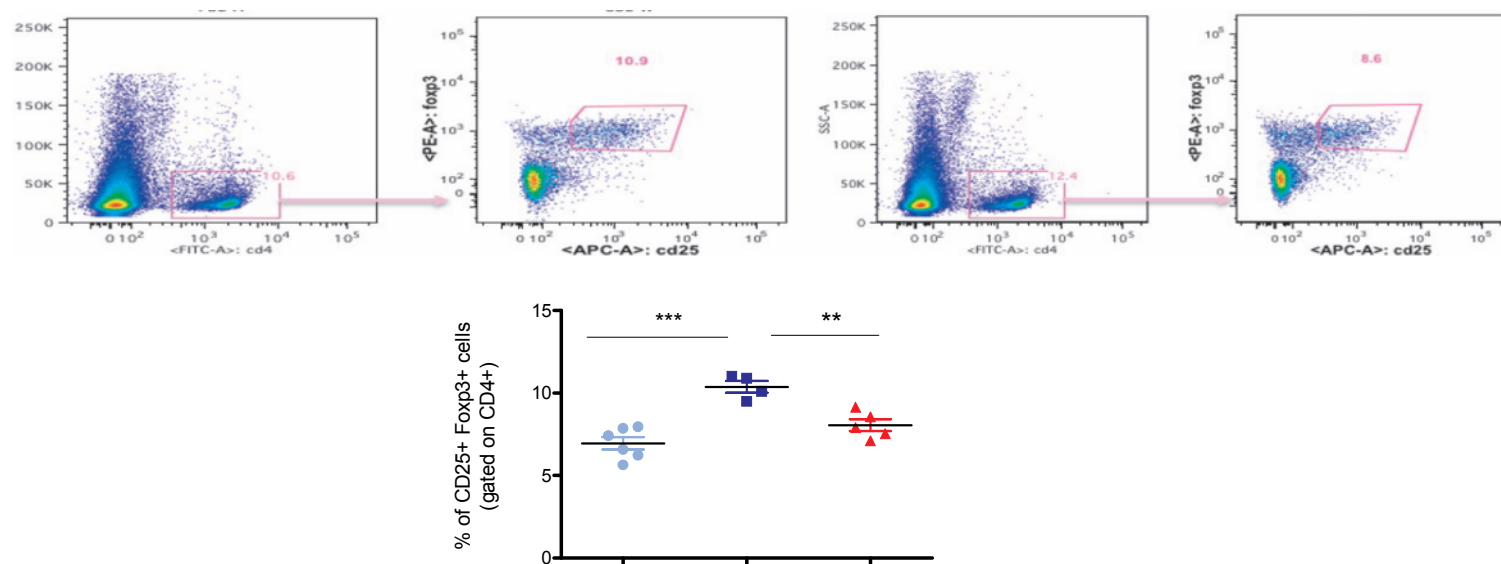**C**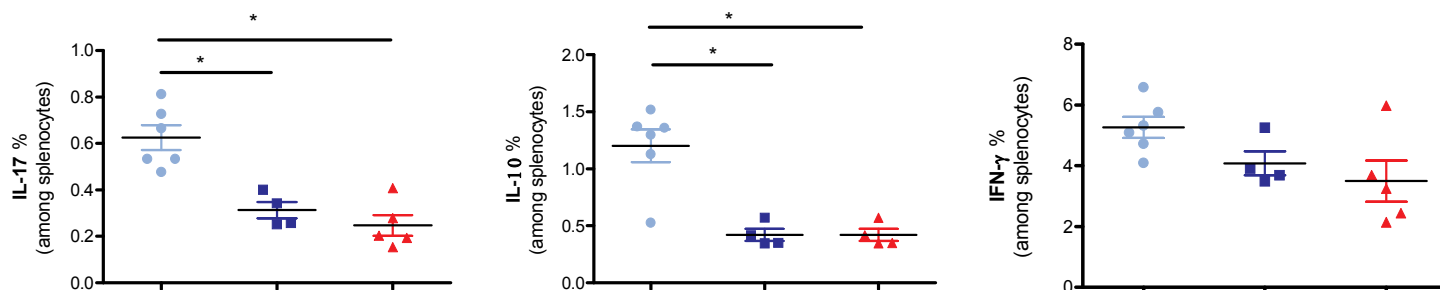**S4 Fig**

Supplement: S4 Fig — A quantitative analysis of flow cytometry staining of blood monocytes (CD11b+CD115+) and subsets, classical (GR1 high) and non classical (GR1 low) monocytes, neutrophils (CD11b+GR-1+), T lymphocytes (CD4+, CD8+) and B lymphocytes (CD19+) cells in Ldlr-/- mice infused with either PBS (n = 5) or Ang II (n = 5) and Ldlr-/-Ido1-/- mice infused with AngII (n = 5) and fed with HFD during 7 days. B representative pictures and quantifications of T regulatory cells (CD25+Foxp3+) gated on CD4+ cells. C quantitative analysis of flow cytometry-based intracellular staining of interleukin (IL)-17, IL-10 and interferon (IFN)-γ gated on splenocytes in the 3 groups of mice. (PDF) [file pone.0193737.s004.pdf]

**A**

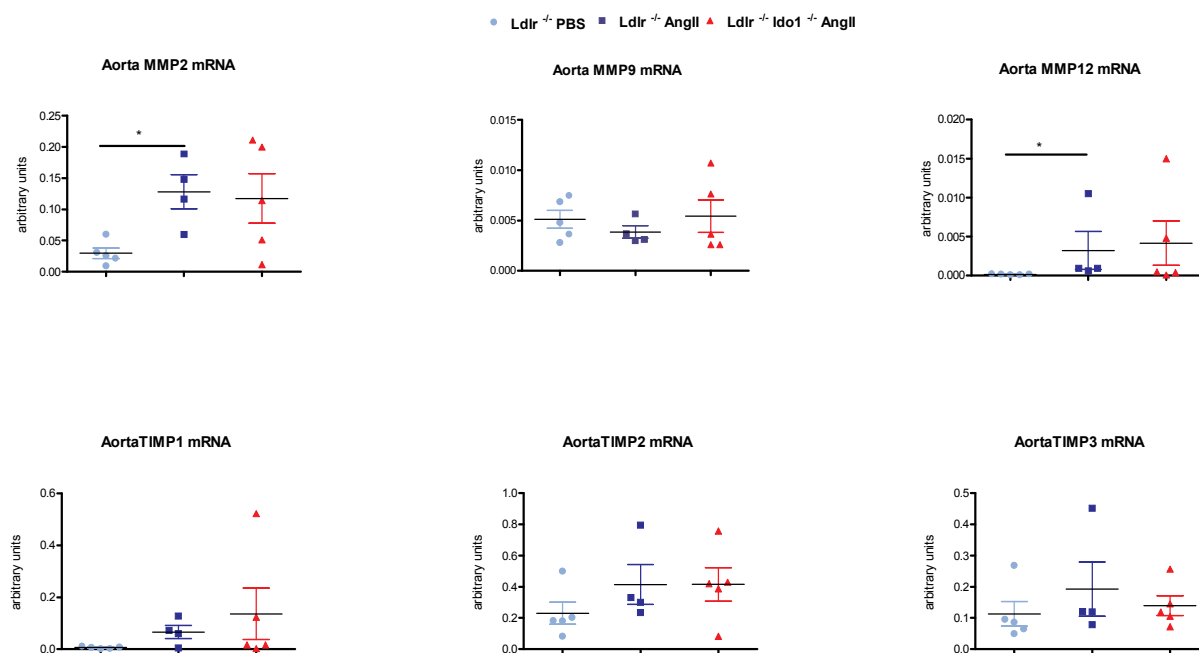

**B**

$Ldlr^{-/-}$  PBS

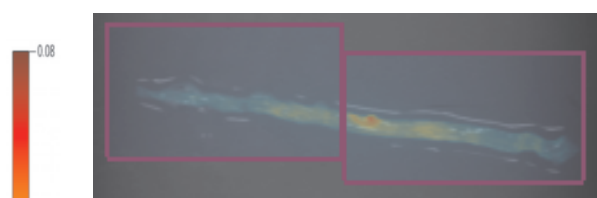

$Ldlr^{-/-}$  AngII

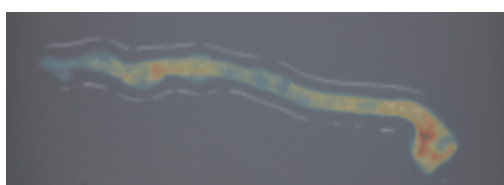

$Ldlr^{-/-}$  Ido-1 $^{-/-}$  AngII

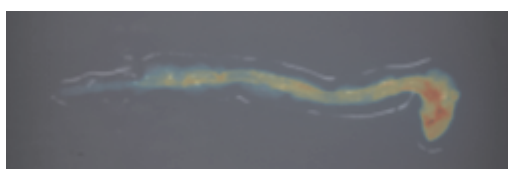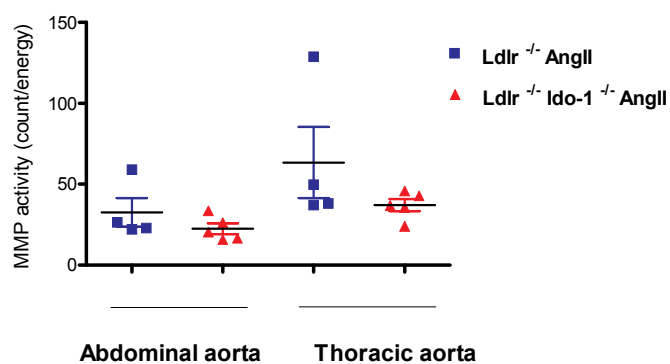

**S5Fig.**

Supplement: S5 Fig — A MMP 2, 9 and 12 and TIMP1, 2, and 3 mRNA in aorta of Ldlr-/- mice infused with either PBS (n = 5) or AngII (n = 5) and Ldlr-/- Ido-1-/- (n = 5) mice infused with Ang II and fed with HFD during 7 days. B Quantification of matrix metalloproteinase (MMP)-sense 680 activity in the abdominal and thoracic aorta, measured by ex vivo reflectance epifluorescence imaging in AngII-infused Ldlr-/- and Ldlr-/- Ido1-/- mice (n = 5/group) fed with HFD during 7days. (PDF) [file pone.0193737.s005.pdf]
